# Anti-candidal Effect of Ocimum sanctum: A Systematic Review on Microbial Studies

**DOI:** 10.7759/cureus.24749

**Published:** 2022-05-05

**Authors:** Chandini R, Saranya R, Khadijah Mohideen, Preethi Nandagopal, Logeswari Jayamani, Sreedevi Jeyakumaran

**Affiliations:** 1 Oral and Maxillofacial Pathology, Sathyabama Dental College and Hospital, Sathyabama Institute of Science and Technology, Chennai, IND; 2 Oral Medicine and Radiology, Sathyabama Dental College and Hospital, Sathyabama Institute of Science and Technology, Chennai, IND; 3 Oral and Maxillofacial Pathology, Meenakshiammal Dental College, Chennai, IND; 4 Oral Medicine and Radiology, Thai Moogambigai Dental College and Hospital, Chennai, IND

**Keywords:** invitro, candida species. antifungal susceptibility. minimum inhibitory concentration, antifungal resistance, ocimum sanctum, anticandidal effect

## Abstract

Candida albicans is the most prevalent candidal species in humans. It is the causative agent and is most commonly associated with more than 90% of serious systemic fungal infections. Even though there are numerous anti-fungal agents, new strains of pathogens develop resistance against these agents. In order to prevent resistance, plant-based drugs can be considered as an alternative therapy. Recent studies show that few herbs consist of active ingredients acting against specific pathogens. The aim of the present study is to understand the anti-candidal effect of *Ocimum sanctum* (Tulsi) based on in-vitro microbial studies. This systematic review followed Preferred Reporting Items for Systematic Reviews and Meta-Analyses Statement Criteria (PRISMA). Articles were collected from the electronic databases of PubMed and Cochrane till 2021. Anti-microbial studies on *O. sanctum* and its action against candidal species were included. We excluded clinical trials, reviews, abstract articles, and interventional studies.

The selected antimicrobial studies used various phytochemical constituents of Tulsi extract, and the anticandidal properties were measured through the zone of inhibition (ZOI). All studies demonstrated the effective anticandidal property of *O. sanctum*, suggesting its possible use as an effective and affordable "adjunct" along with standard care for systemic and topical candidal infections. The main components of *O. sanctum* responsible for anticandidal activity were likely to be eugenol and linalool. However, the mechanism of action of these constituents is unclear. Further research assessing the toxicity, durability, and other assessments followed by clinical trials is necessary to explore the potential of Tulsi in combating oral conditions.

## Introduction and background

Candida species are the most common organisms involved in topical and systemic fungal infections [1]. *Candida albicans* is the most common and virulent among the candidal species [2,3]. There are numerous anti-fungal agents, but new strains of pathogens develop resistance against these agents. In order to prevent this resistance, plant-based drugs can act as an alternative therapy. Herbal treatments are common among urban and rural communities of the Indian population. Recent studies show that these herbs consist of active ingredients acting against specific pathogens [4].

*Ocimum sanctum* (Tulsi) is a highly regarded culinary and medicinal aromatic herb from the Lamiaceae family, indigenous to the Indian subcontinent, and which has been utilized in Ayurvedic medicine for more than 3000 years. For its medicinal properties, Tulsi is regarded as an "Elixir of Life" and has been used to treat various ailments. Tulsi leaf extracts are used to treat bronchitis, rheumatism, and pyrexia [1]. Treatment of epilepsy, asthma or dyspnea, hiccups, cough, skin and hematological illnesses, parasite infections, neuralgia, headache, wounds, and inflammation [2], and oral problems [3] are among the other known medicinal uses. Pharmacological and clinical studies showed that *O. sanctum* consists of terpenoids, iridoids, [5] phenolic compounds, and flavonoids. These active components act as anti-microbial, anti-oxidative, anti-inflammatory, anti-arthritic, and anti-pyretic agents. Only a few studies use *O. sanctum* as an anti-fungal agent [1,6]. The essential oils derived from these specific plants are targeted against the ergosterol biosynthesis pathway. These are the active end products of cell wall synthesis. Most of the phytochemical constituents of *O. sanctum* alter the fungal organism's structural and functional integrity [7].

A systematic evaluation of studies on using Tulsi as part of a polyherbal formulation against candidal species was conducted through in vitro studies. This study aimed to summarise and critically evaluate Tulsi to assess the current evidence on the herb's efficacy against candidal species.

Aim and objectives

This systematic review aimed to determine the role of *O. sanctum* and its anti-candidal action through in vitro microbial studies.

## Review

Methodology

We searched for the literature that used *O. sanctum* as a key element against candidal species in microbial studies from 2003 to 2021 in the Cochrane and PubMed databases.

Search strategy

Keywords and Search Terms

The keywords in the review question were identified based on the SPIDER framework, Phenomenon of Interest, Design, and Evaluation. The keywords were discovered, and a list of synonyms and MeSH terms was developed to search. A Boolean operator search was established, and MeSH terms were as follows: (Anticandidal effect AND Ocimum sanctum); (Ocimum sanctum AND Candida Species); (Ocimum sanctum AND Anti-fungal effect); and (In vitro candida studies AND Anti-fungal Ocimum sanctum).

Inclusion and Exclusion Criteria

Based on the search results from PubMed and the Cochrane database, 352 articles were identified. The articles published in English from 2003 to 2022 were included. This review included original research, in-vitro studies, that were based on using *O. sanctum* as a constituent to act against candidal species. The articles in other languages, case reports, abstracts-only articles, randomized control trials, duplicates, and letters to the editor were not included. The articles were examined based on the eligibility criteria, and the data were extracted. We also excluded the data that was used in clinical studies, and finally, eight articles were selected for the review.

Review Process

All the reviewers were involved in the entire review process. This review process included the various stages of screening, data extraction, and observations in the study. The articles obtained by the Boolean search were screened for abstracts, and titles that met the inclusion criteria were organized.

Results

**Figure 1:**
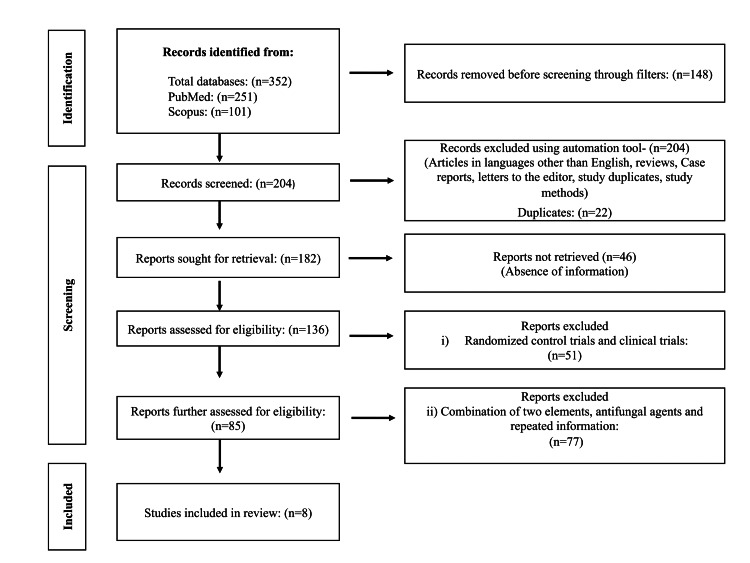
PRISMA protocol - systematic review for included articles

After the database search, a total of 352 articles were identified for this systematic review. One hundred and forty-eight records were considered ineligible by using filters before screening through filters. Later, 204 records were removed using an automation tool for abstracts and duplicates. Finally, eight articles were carefully chosen and included in the review process. The full text of eight articles that met the criteria was assessed. Additionally, the Preferred Reporting Items for Systematic Review and Meta-Analysis (PRISMA) 2020 was used in the process (Figure 1).

Quality Assessment and Risk of Bias Criteria

The Critical Appraisal Skills Programme (CASP; CASP International Network, 2013) systematic review checklist was used to evaluate the study articles by the reviewers. 

The quality and the risk-of-bias tool for the selected studies were assessed through the United States national toxicology program for in vitro studies, which is performed based on seven domains: (i) same experimental condition, (ii) blinding during the study, (iii) incomplete data, (iv) exposure characterization, (v) outcome assessment, (vi) reporting, and (vii) other.

Risk of Bias Assessment of Included Studies

The study was judged based on "low" and "moderate" risk, and "no information" was given to the study, which had no answers according to the domain assessed (Table 1). Out of eight articles, one article showed a low risk of bias and seven articles showed a moderate risk of bias. These studies were reported under the guidelines of risk assessment for in-vitro studies developed by the United States national toxicology program.

**Table 1:** Risk of bias assessment of in vitro studies Judgement; + = low risk, − = moderate risk, ? = no information

Study details	Same experimental condition D1	Blinding during the study D2	Incomplete data D3	Exposure characterization D4	Outcome assessment D5	Reporting D6	Other D7	Overall
Hemaiswarya et al. [8]	+	+	+	+	+	+	+	+
Khan et al. [9]	+	+	+	+	−	−	−	−
Khan et al. [10]	−	+		+	+	?	+	−
Gopalkrishna and Muddaiah [11]	−	+	+	+	−	−	−	−
Patil et al. [12]	+	?	+	+	?	−	−	−
Zaidi et al. [13]	−	+	−	−	−	−	+	−
Janani et al. [14]	?	+	+	+	+	+	+	−
Prajapati et al. [15]	+	?	+	−	−	−	+	−

Data Extraction

The records were screened by all the authors, and the following data were collected: title, authors, journal details, study design, phytochemical constituents, method of extraction, synergistic action, mechanism of action, target organism, minimum inhibitory concentration (MIC), zone of inhibition (ZOI), and overall study result (Table 2). The eligible articles were categorized, and the data were extracted and analyzed. These studies also focus on the mechanism of action and synergetic effect of *O. sanctum* against candida. The anti-candidal effects were measured by the ZOI and MIC.

**Table 2:** Summary of study articles

Study	Study design	Phytochemical constituents	Method of extraction	Synergistic action	Mechanism of action	Target organism	Minimum inhibitory concentration (%)	Zone of inhibition (mm)	Study result
Hemaiswarya et al. [8]	In vitro	Eugenol	Aqueous and disk diffusion method	Amphotericin B	Attacks the cell membrane and results in cell lysis	C. albicans	-	-	*O. sanctum* + Amphotericin B = increased antifungal effect
Khan et al. [9]	In vitro	Eugenol	Hydrodistillation	-	Target the structural and functional integrity of the cytoplasmic membrane in Candida	*C. albicans*, *C. tropicalis*, *C. glabrata*, *C. parapsilopsis*, *C. krusei*	0.015-0.03, 0.015-0.03, 0.02-0.03, 0.02-0.05, 0.03-0.045	-	Anti-fungal activity and minimal toxicity toward the human erythrocytes
Khan et al. [10]	In vitro	Ergosterol methyl eugenol, linalool, 1,8-cineole (eucalyptol), carvacrol, methyl chavicol and caryophyllene	Aqueous and disk diffusion method	Aloe vera gel	Inhibit H+ extrusion through ATPase	All candida species	C. albicans - 42% C. tropicalis - 48%	-	Linalool had a more efficient anti-candidal effect
Gopalkrishna et al. [11]	In vitro	Sterol of *O. sanctum*	Aqueous and disk diffusion method	Centratherum anthelminticum	Inhibition	*C. albicans*, *C. krusei*, *C. tropicalis*, *C. parapsilosis*, *C. glabrata* and *C. dubliniensis*	60.00 to 75.7, 53.3 to 73.7, 45.7 to 57.00, 59.00 to 69.3, 54.7 to 67.00 and 44.7 to 45.6	75.7 ± 4.33, 73.7 ± 0.88, 57.0 ± 0.0, 69.3 ± 3.48, 67.0 ± 1.00, 45.7 ± 1.33	*C. anthelminticum* and *O. sanctum* seed oils - active against oral pathogens
Patil et al. [12]	In vitro	Alkaloid, flavonoids, glycosides, saponins, steroids, tannins, anthraquinones, phenolic compounds	Disk diffusion method-aqueous extract ethanol, methanol, and acetone extract	*Tinospora cordifolia*, *Azadirachta indica*	Inhibition	C. albicans	-	Aqueous- nil Ethanol-14 Methanol-18 Acetone-18	*Tinospora cordifolia*, *Azadirachta indica*, and *O. sanctum* leaves extract is an efficient anti-fungal agent
Zaidi et al. [13]	In vitro	Alkaloids carbohydrates glycosides phenolics compounds and tannins proteins amino acids flavonoids terpenoids saponins phlobatannins steroids	Methanol extract, benzene extract, aqueous extract	Fluconazole	Inhibitory action and cell lysis	C. albicans	16 mg/ml- High vaginal swab 32 mg/ml- Urine and 64 mg/ml- Catheter tip	Leaf -32 Fluconazole- 16 Leaf with fluconazole-04 Stem- 16 Fluconazole- 16 Stem with fluconazole-04	*O. sanctum* leaf with fluconazole showed more antifungal activity - HVS,U,CT
Janani et al. [14]	In vitro	Silver nanoparticles	UV-visible spectrometric analysis	Fluconazole and *Justicia adhatoda*	Inhibitory action and cell lysis	C. albicans	-	16	*O. sanctum* and *J. adhatoda* formulation mediated silver nanoparticles exhibit significant anti-fungal activity with minimal side-effects
Prajapati et al. [15]	In vitro	Sterol of *O. sanctum*	Agar disk diffusion method	SJ: *Syzygium jambolanum*, FR: *Ficus religiosa*, AC: *Allium cepa*, TO: *Thuja occidentalis*, HA: *Holarrhena antidysentery*, EG: *Eucalyptus globulus*	Inhibition	C. albicans	-	SJ- 285 FR- 97 OS- 60 AC- 137 TO-205 HA- 0 EG- 89	The effectiveness of zone inhibition against the growth of pathogenic fungi *C. albicans* are *S. jambolanum* > *T. occidentalis* > *A. cepa* > *F. religiosa* > *E. globulus* > *O. sanctum* > *H. antidysenterica*

Data Analysis and Interpretation

All the studies included from the years 2003 to 2021 were considered. Eight articles met the criteria, and all were in-vitro studies that used *O. sanctum* as a principal constituent against candidal species. All the included studies had phytochemical constituents of *O. sanctum*, which were extracted through different methods and targeted different Candida species. The results of in vitro on the combination of *O. sanctum* with other elements and their synergetic action were analyzed (Table 3).

**Table 3:** Results of in vitro on the combination of Ocimum sanctum with other elements

Study	Candida Species	Chemical constituents	Combined therapy	Result
Hemaiswarya et al. [8]	C. albicans	Ergosterol	Amphotericin B	Synergetic effect with fractional inhibitory concentration < 0,5
Khan et al. [10]	*C. albicans,* *C. tropicalis*	Ergosterol methyl eugenol, linalool, 1,8-cineole (eucalyptol), carvacrol, methyl chavicol and caryophyllene	Aloe vera gel	Additive effect
Gopalkrishna et al. [11]	*C. albicans*, *C. krusei*, *C. tropicalis*, *C. parapsilosis*, *C. glabrata* and *C. dubliniensis*	Sterol of *O. sanctum*	Centratherum anthelmintic	The synergetic effect with fractional inhibitory concentration is higher
Patil et al. [12]	C. albicans	Alkaloid flavonoids glycosides saponins steroids tannins anthraquinones phenolic compounds	*Tinospora cordifolia*, *Azadirachta indica*	Synergetic effect with fractional inhibitory concentration < 0,5
Zaidi et al. [13]	C. albicans	Alkaloids carbohydrates glycosides phenolics compounds and tannins proteins amino acids flavonoids terpenoids saponins phlobatannins steroids	Fluconazole	Synergetic effect with fractional inhibitory concentration- 300 fold increase
Janani et al. [14]	C. albicans	Silver nanoparticles	Fluconazole and *Justicia adhatoda*	Additive effect
Prajapati et al. [15]	C. albicans	Sterol of *O. sanctum*	SJ: *Syzygium jambolanum*, FR: *Ficus religiosa*, AC: *Allium cepa*, TO: *Thuja occidentalis*, HA: *Holarrhena antidysentery*, EG: *Eucalyptus globulus*	Additive effect

Discussion

Most of the candidal species are harmless commensals, where 70% of the population resides in the genito-urinary tract and the aerodigestive tract. Overgrowth of these commensal species can cause disease. Over 75% of women suffer from vulvovaginal candidiasis and 4% from nosocomial infections, which cause serious systemic illness leading to death [16]. Antifungal drugs act on the organism by disrupting the cell membrane, inhibiting mitosis, and preventing DNA synthesis [17]. The prolonged use of commercially available antifungal drugs creates a negative effect on human health by developing resistance to drugs, which has become challenging to treat the disease [6,18]. Hence, the search for natural phytochemicals isolated from plants is considered a good alternative to synthetic chemicals.

Study data have demonstrated that a variety of phytochemicals derived from natural sources enhance the activity of antifungal drugs by reversing the fungi's resistance activity and inhibiting the active efflux of antifungal agents across the plasma membrane. For a specific strain, an antifungal drug interacts synergistically with herbal extracts. The goal of this study was to determine the ability of Tulsi extracts to suppress Candidal growth [19].

Holy basil extract was tested for antimicrobial activity using minimum inhibitory concentration and zone of inhibition levels against *Candida albicans* in human dental plaque. The findings demonstrated antifungal activity at concentrations of 0.015-0.03% or at a higher level of *O. sanctum* [10]. The investigation confirmed that a zone of inhibition of 22 mm was produced at a concentration of 6% holy basil extract, which was the broadest zone of inhibition recorded among the 10 concentrations studied. The zone of inhibition surrounding the positive control was 25 mm, while there was none around the negative control [20]. Also, Gopalkrishna et al. found that antifungal activity on *C. albicans* is increased with an increase in the concentration of *O. sanctum* [11]. They observed that *Centratherum anthelminticum*, belonging to a relative of the sunflower family, produces a synergistic effect that relatively increases the inhibitory zone with the increase in the concentration of Tulsi powder on *C. albicans*.

Avetisyan et al. while analyzing the constituents of *O. sanctum* (essential oils) by gas chromatography/mass spectrometry (GC/MS) found that 1, 8-cineole and β-bisabolen, and eugenol were the principal constituents to act as immunostimulants [21]. In their clinical trials, Rajalakshmi et al. and Das et al. reported that viral encephalitis patients were treated with 10 mg of Tulsi extract and had a better survival rate compared to the dexamethasone dose, which was administered to a different group [21,22]. These findings demonstrate the enhanced antibody response and neutrophil activity of *O. sanctum* extract against three pathogens: *Escherichia coli*, *Staphylococcus aureus*, and *C. albicans*.

Singh et al. described the impact of *O. sanctum* action against oral candidiasis with its two main components, eugenol and linalool, in which linalool proved to have the better antifungal property. Khan et al. [9,10] in their experiments demonstrated the anti-fungal nature of Tulsi and its synergistic action with azoles. In this experiment, fungal strains, both fluconazole-resistant and non-resistant, were taken and treated with *O. sanctum* L. extract (methyl chavicol and linalool). Finally, the extracts were synergistic with fluconazole and active against strains of *C. albicans*, *C. tropicalis*, *C. glabrata*, *C. parapsilosis*, and *C. krusei* [23].

Limitations

Several issues were identified in the literature to address the efficacy of *O. sanctum* against fungal species. As a recent concern has arisen with respect to the development of azole-resistant fungal species leading to chronic suppressive therapy, new research should focus on the fungicidal capabilities to assess the compliance of *O. sanctum* with other components to produce an effective outcome. Additionally, the cost-effectiveness of the traditional herb can create a major impact on the therapy selection and treatment outcome. This will allow more unbiased estimates of the relative risk of resistance that is associated with different treatment regimens. Research on the effectiveness of alternative and complementary medicine and therapies, for both prophylaxis and treatment, should be considered in the future. A large-scale multidisciplinary approach using clinical trials will aid in giving clear evidence to confirm the antimicrobial and antifungal actions.

## Conclusions

The evidence of prophylactic efficacy of *O. sanctum* was good but insufficient to draw conclusions about its therapeutic use as the mechanism of action, clinical outcomes, and adverse effects were unclear. The available evidence suggests that a combination of *O. sanctum* with azoles can improve performance and thereby decrease anti-fungal consumption. Long-term evaluation is necessary to show the effect of the *O. sanctum* combination of anti-fungal drugs on patient and economic outcomes, including mortality. New research in the field is mandatory to indicate that *O. sanctum* is a viable alternative to other fungicides and can be used as a drug to combat infection. Future research in this area should include RCTs with long-term follow-ups to guide the development of *O. sanctum* combined drug guidelines to overcome some of the weaknesses found in the literature.
